# Supplementary figures and images for: NAD kinase promotes Staphylococcus aureus pathogenesis by supporting production of virulence factors and protective enzymes
Source: eLife. 2022 Jun 20;11:e79941. doi: 10.7554/eLife.79941 (PMC9208755; doi:10.7554/eLife.79941)

Figure supplement 1A

Original

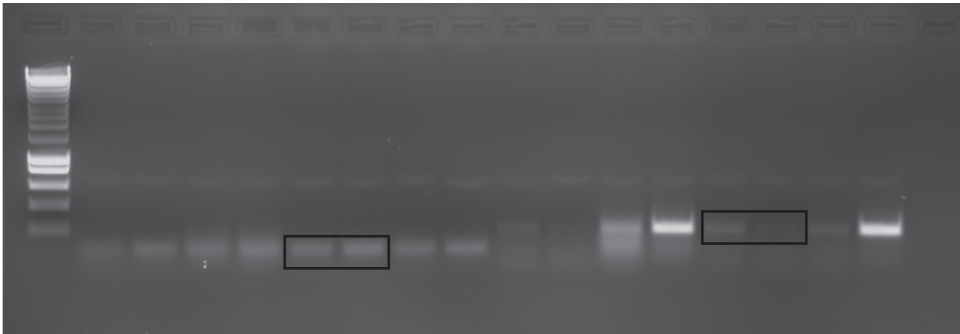

Cropped

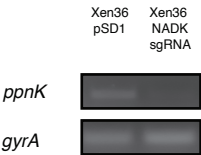

Supplement: Figure 1—figure supplement 1—source data 1. [file elife-79941-fig1-figsupp1-data1.pdf]

Figure 1-supplement 1B

Original

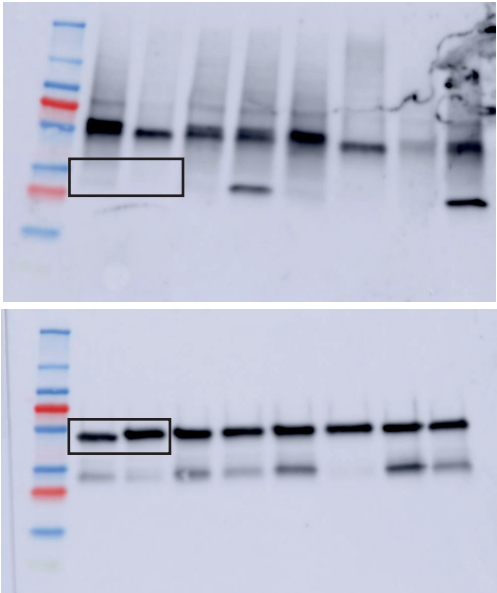

Cropped

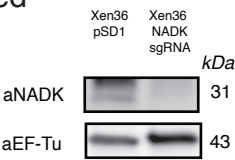

Supplement: Figure 1—figure supplement 1—source data 2. [file elife-79941-fig1-figsupp1-data2.pdf]

Figure supplement 1E

Original

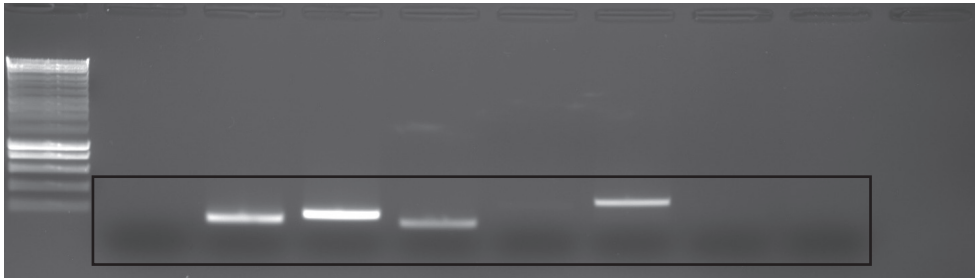

Cropped

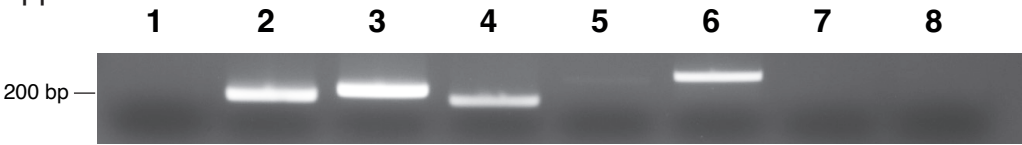

Supplement: Figure 1—figure supplement 1—source data 3. [file elife-79941-fig1-figsupp1-data3.pdf]

Figure supplement 1F

Original

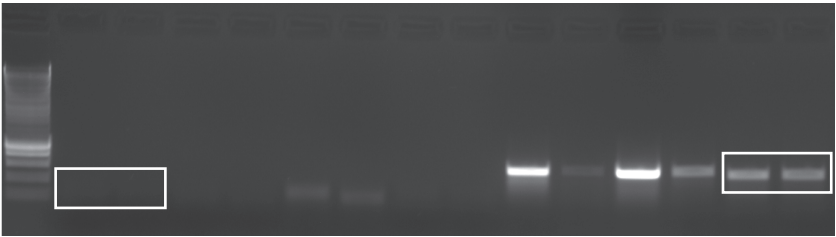

Cropped

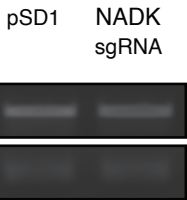

Supplement: Figure 1—figure supplement 1—source data 4. [file elife-79941-fig1-figsupp1-data4.pdf]

Figure 4D

Original

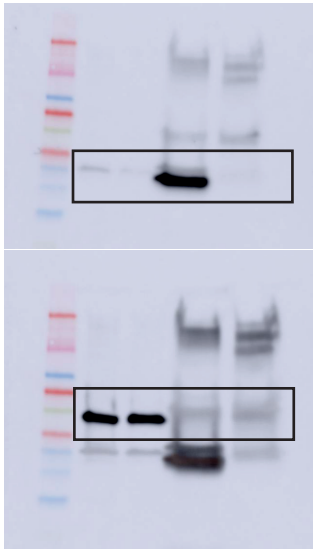

Cropped

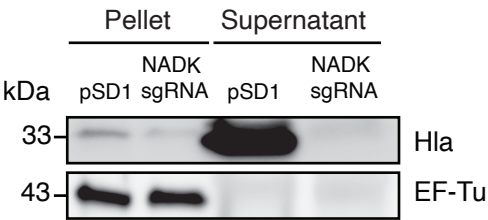

Supplement: Figure 4—source data 1. [file elife-79941-fig4-data1.pdf]

**Figure 4F**  
Original

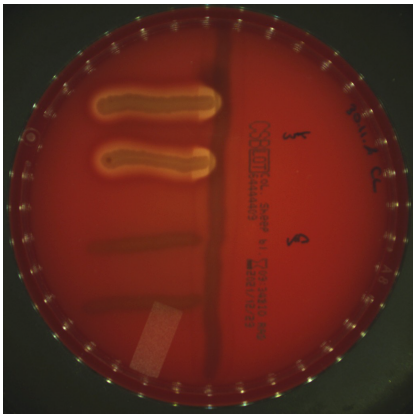

Cropped

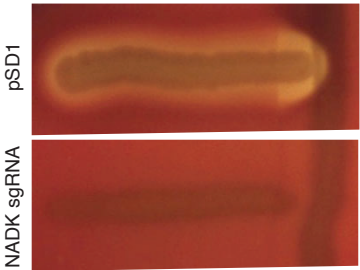

Supplement: Figure 4—source data 2. [file elife-79941-fig4-data2.pdf]

**Figure 5A**  
Original

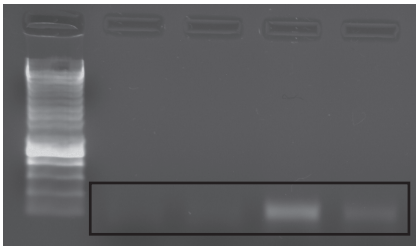

Cropped

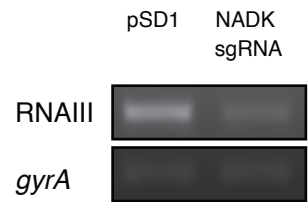

Supplement: Figure 5—source data 1. [file elife-79941-fig5-data1.pdf]

**Figure 5B**  
Original

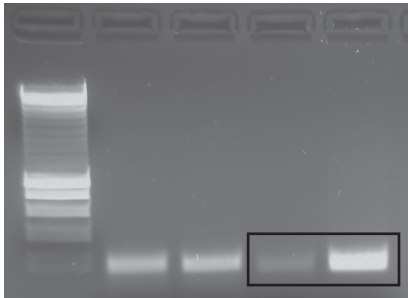

Cropped

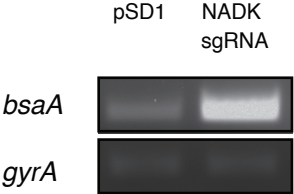

Supplement: Figure 5—source data 2. [file elife-79941-fig5-data2.pdf]

**Figure 5C**  
Original

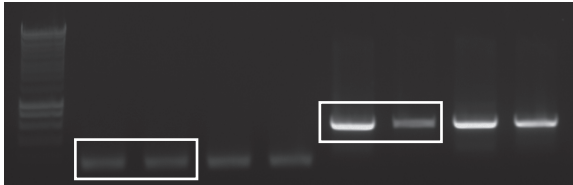

Cropped

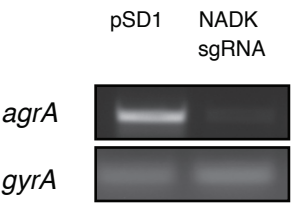

Supplement: Figure 5—source data 3. [file elife-79941-fig5-data3.pdf]
