# Supplementary material for: NAD kinase promotes Staphylococcus aureus pathogenesis by supporting production of virulence factors and protective enzymes
Source: eLife. 2022 Jun 20;11:e79941. doi: 10.7554/eLife.79941 (PMC9208755; doi:10.7554/eLife.79941)
Supplement: Supplementary file 2. [file elife-79941-supp2.docx]

| Uniprot | Protein | Description | Log2R* | P** |
| --- | --- | --- | --- | --- |
| Q2FV54  Q2G000  Q2G261  Q2FZZ3  Q2G0D9  Q2G280  P0A086  Q2FVL7  Q2G0E0  Q2FYU7 | OatA  Trx2  SodM  -  GraS  -  MsrA2  -  GraR  KatA | O-acetyl transferase  Thioredoxin 2  Superoxide dismutase [Mn/Fe]  Thioredoxin domain-containing protein  Sensor histidine kinase  Peroxiredoxin  Methionine sulfoxide reductase  Thioredoxin domain-containing protein  Response regulator protein  Catalase | 3.07  1.79  1.68  1.59  1.31  1.26  1.10  1.02  1.00  0.59 | 1.78E-05  2.75E-05  2.11E-06  7.22E-04  4.00E-04  6.69E-07  2.25E-05  1.94E-03  2.23E-03  1.00E-05 |

* Log2R=Log2[pSD1]/[NADK sgRNA]

**Adjusted p value
