# Supplementary material for: NAD kinase promotes Staphylococcus aureus pathogenesis by supporting production of virulence factors and protective enzymes
Source: eLife. 2022 Jun 20;11:e79941. doi: 10.7554/eLife.79941 (PMC9208755; doi:10.7554/eLife.79941)
Supplement: Supplementary file 4. [file elife-79941-supp4.docx]

| Uniprot | Protein | Description | Log2R* | P** |
| --- | --- | --- | --- | --- |
| Q2G000  P0A0J3  Q2FYU7  Q2FKL3  Q2FZW4  O05204  Q2FXI6  Q2G280 | -  SodA  KatA  Tpx  DltC  AhpF  -  - | Thioredoxin domain-containing protein  Superoxide dismutase [Mn]  Catalase  Thiol peroxidase  D-alanyl carrier protein  Alkyl hydroperoxide reductase subunit F  Thioredoxin domain-containing protein  Peroxiredoxin | +  +  +  +  +  +  +  + | NA  NA  NA  NA  NA  NA  NA  NA |

* Log2R=Log2[pSD1]/[NADK sgRNA]; +: protein detected in pSD1 strain and not detected from NADK sgRNA strain

**Adjusted p value: NA: not applicable
