# Supplementary material for: NAD kinase promotes Staphylococcus aureus pathogenesis by supporting production of virulence factors and protective enzymes
Source: eLife. 2022 Jun 20;11:e79941. doi: 10.7554/eLife.79941 (PMC9208755; doi:10.7554/eLife.79941)
Supplement: Supplementary file 6. [file elife-79941-supp6.docx]

| Uniprot | Protein | Description | Log2R* | P** |
| --- | --- | --- | --- | --- |
| Q2FVK1  Q2FWM8  Q2FWM4  Q2FVK2  P0C818  P0C7Y1  Q2FUU5  Q2G1X0  Q2FVK3  Q2FWP0  Q2G2R8  Q2FWN9 | HlgB  Hld  AgrA  HlgC  PsmA4  PsmA1  Lip1  Hly  HlgA  LukL1  SspP  LukL2 | Gamma-hemolysin component B  Delta-hemolysin  Accessory gene regulator protein  Gamma-hemolysin component C  Phenol-soluble modulin alpha 4  Phenol-soluble modulin alpha 1  Lipase  Alpha-hemolysin  Gamma-hemolysin component A  Leukocidin-like protein 1  Staphopain A  Leukocidin-like protein 2 | +  +  +  +  +  +  +  7.99  5.59  3.35  3.27  2.98 | NA  NA  NA  NA  NA  NA  NA  2.92E-09  1.60E-06  7.16E-06  1.17E-06  2.56E-07 |

*Log2R=Log2[pSD1]/[NADK sgRNA]; +: protein detected in pSD1 strain and not detected from NADK sgRNA strain

**Adjusted p value: NA: not applicable
